# Endocarditis due to *Staphylococcus lugdunensis*—a retrospective national registry–based study

**DOI:** 10.1007/s10096-020-04134-w

**Published:** 2020-12-30

**Authors:** Malin Hagstrand Aldman, Magnus Rasmussen, Lars Olaison, Lisa I. Påhlman

**Affiliations:** 1 Department of Clinical Sciences Lund, Division of Infection Medicine, Lund University, Lund, Sweden; 2 Division of Infectious Diseases, Skåne University Hospital Lund, Lund, Sweden; 3 Department of Infectious Diseases, Sahlgrenska University Hospital, Institute of Biomedicine, University of Gothenburg, Gothenburg, Sweden; 4 Swedish Registry of Infective Endocarditis, Swedish Society of Infectious Diseases, Gothenburg, Sweden; 5 Wallenberg Centre for Molecular Medicine, Lund University, Lund, Sweden

## Abstract

We present characteristics of infective endocarditis (IE) caused by *Staphylococcus lugdunensis* and compare with IE caused by *Staphylococcus aureus* and other CoNS, in the National Swedish Registry of IE (2008–2018). Thirty episodes of *S. lugdunensis* IE were registered, of which 21 cases affected native valves, and 7 patients were subjected to surgery. The mortality rate at 30 days was significantly higher for *S. lugdunensis* IE (20%, *n* = 6), than for IE caused by other CoNS (7%) or *S. aureus* (9%) *p* = 0.016. Septic embolisation was only reported in two cases (7%). The most common treatment was isoxazolyl penicillin (*n* = 18).

## Introduction

*Staphylococcus lugdunensis* belongs to the group of CoNS and was first described in 1988 [1–3]. Since then, *S. lugdunensis* has attracted increasing interest as a cause of infective endocarditis (IE). It is known to infect native valves to a larger extent than other CoNS, and to have a clinical presentation more similar to *S. aureus* [4]. Several case reports indicate that *S. lugdunensis* can cause severe IE with rapid destruction of heart valves and massive septic embolisation [5–9], and many studies have emphasised the importance of early surgical intervention [7, 8, 10]. *S. lugdunensis* is also often susceptible to narrow-spectrum antibiotics including penicillin, and recent reports have indicated that benzyl penicillin may be a better alternative for susceptible isolates than standard treatment with isoxazolyl penicillin or vancomycin [11–13].

Our aim was to describe the clinical presentation of IE caused by *S. lugdunensis*, and to compare it with the clinical presentation of IE caused by other CoNS and *S. aureus*. A secondary aim was to present the antibiotic treatment of *S. lugdunensis* IE.

## Material and methods

We retrospectively reviewed cases of IE caused by *S. lugdunensis*, *S. aureus* and other CoNS from the Swedish Registry of Infective Endocarditis between 2008 and 2018.

Cases were classified according to Duke’s criteria [14, 15]. If information about comorbidities were missing, we interpreted it as a negation of that condition. For other missing data, no imputations were made. Treatment delay was defined as days from onset of symptoms until start of IE treatment. Definite antibiotic treatment was defined as the antibiotic given more than 50% of the treatment time. Antibiotic susceptibility data were received by personal contact with the local microbiological department at each site. Antibiotic susceptibility testing was performed according to EUCAST guidelines [16].

Comparisons between groups were made with chi-squared test when testing categorical variables and Mann-Whitney *U* test for continuous data. Survival data were calculated with a Kaplan-Meier survival curve and log-rank test. Two-tailed *p* < 0.05 was regarded as statistically significant. Analyses were performed using the SPSS software, version 25 (SPSS, Armonk, NY, USA).

The study was approved by the Medical Ethics Committee (Institutional Review Board) of Lund University (Dnr 2017/1002).

## Results and discussion

In total, we found 30 cases of IE caused by *S. lugdunensis*, 262 cases of IE caused by other CoNS and 1892 cases of IE caused by *S. aureus.* Clinical characteristics of the different groups are summarised in Table 1. Patients with *S. lugdunensis* IE were significantly older than patients with IE caused by *S. aureus* (73 vs 66 years, *p* = 0.01). When iv-drug users were excluded, the median age of *S. aureus* cases increased to 69.5 years, and the age difference between the groups was no longer significant (*p* = 0.44). In agreement with previous reports, 21 patients (70%) in the *S. lugdunensis* group had native valve IE, and the most common localisation was the aortic valve (60%) [7, 17]. The proportion of native valve IE in the *S. lugdunensis* group was similar to that in *S. aureus* IE, but significantly higher than for other CoNS (70% vs 35%, *p* = 0.0001).

**Table 1:** Patient characteristics and outcome data

Bacteria (*n*)	*S. lugdunensis* *n* = 30	CoNS *n* = 262	*P* value CoNS *vs S. lugdunensis*	*S. aureus* *n* = 1892	*P* value *S. aureus vs S. lugdunensis*
Background data					
Age (years); median (IQR)	*73 (65–84)*	72 (61–80)		66 (45–79)	*p* = 0.01
Gender-female	*11 (37%)*	86 (33%)		725 (38%)	
Diabetes	*9 (30%)*	58 (22%)		349 (18%)	
Cancer last 5 years	*6 (20%)*	46 (18%)		173 (9%)	*p* = 0.042
IV drug users	*0 (0%)*	9 (3%)		448 (24%)	*p* = 0.002
Prosthetic valve	*8 (27%)*	115 (44%)		255 (14%)	*p* = 0.037
Pacemaker/ICD	*1 (3%)*	74 (28%)	*p* = 0.031	324 (17%)	*p* = 0.046
Native valve disease	*5 (17%)*	55 (21%)		222 (12%)	
Treatment delay, days median (IQR)	*9 (4-15)*	10 (3–26)		5 (2-9)	*p* < 0.001
Dukes criteria					
Definite	*27 (90%)*	194 (74%)		1544 (82%)	
Possible	*3 (10%)*	67 (26%)		338 (18%)	
Localisation					
Aortic	*18 (60%)*	121 (46%)		577 (31%)	*p* = 0.001
Mitral	*10 (33%)*	76 (29%)		596 (32%)	
Tricuspid	*1 (3%)*	22 (8%)		441 (23%)	*p* = 0.01
Type of infection					
Prosthetic IE	*6(20%)*	110 (42%)	*p* = 0.02	245 (13%)	
Pacemaker/ ICD IE	*1(3%)*	48 (18%)	*p* = 0.01	179 (9%)	
Native valve IE	*21 (70%)*	90 (35%)	*p* = 0.0001	1103 (58%)	
Community acquired	*25 (83%)*	179 (68%)		1543 (82%)	
Outcome					
Antibiotic treatment, median days (IQR)	*31 (18–37)*	35 (28–42)	*p* = 0.046	30 (28-40)	
Embolisation	*2 (7%)*	62 (24%)	*p* = 0.033	907 (48%)	*p* < 0.001
Surgical intervention	*7 (23%)*	111 (42%)	*p* = 0.044	455 (24%)	
Day of surgery, median (IQR)	*5* *(1–9)*	12 (5-20)		12 (7-23)	
Mortality at 30 days In-hospital mortality	6 (20%)* *7 (23%)*	17(7%) 49 (19%)		166 (9%) 268 (14%)	
Day of death in hospital, Median (IQR)	*9 (8–23)*	36 (28–47)	*p* = 0.007	25 (14-39)	*p* = 0.016

Data are presented as number and (%) unless otherwise stated. Survival data calculated with Kaplan-Meier survival curve and log-rank test

*ICD* intracardiac device, *IQR* interquartile range

*p = 0.016

There was a significantly lower occurrence of septic embolisation in patients with *S. lugdunensis* IE (7%, *n* = 2), both compared with the *S. aureus* group (48%, *p* < 0.001) and the other CoNS group (24%, *p* = 0.033). This is in contrast to earlier published studies, reporting a high frequency of severe septic embolisation [5, 6, 8, 9]. This discrepancy could be a result of previous publication bias, unthorough clinical examination or failure to report correctly to the database registry. Speaking against the latter is that the embolisation frequency for *S. aureus* was in line with previously published data [18].

Moreover, earlier studies have reported a need of surgical intervention in a large proportion of cases [5, 7, 19], but only seven patients with *S. lugdunensis* IE (23%) underwent surgery in our cohort. This was similar to the *S. aureus* group (24%) but lower than for other CoNS cases (42% vs 23%, *p* = 0.044), which can probably be attributed to the high proportion of prosthetic valve IE in the other CoNS group.

**Fig. 1:**
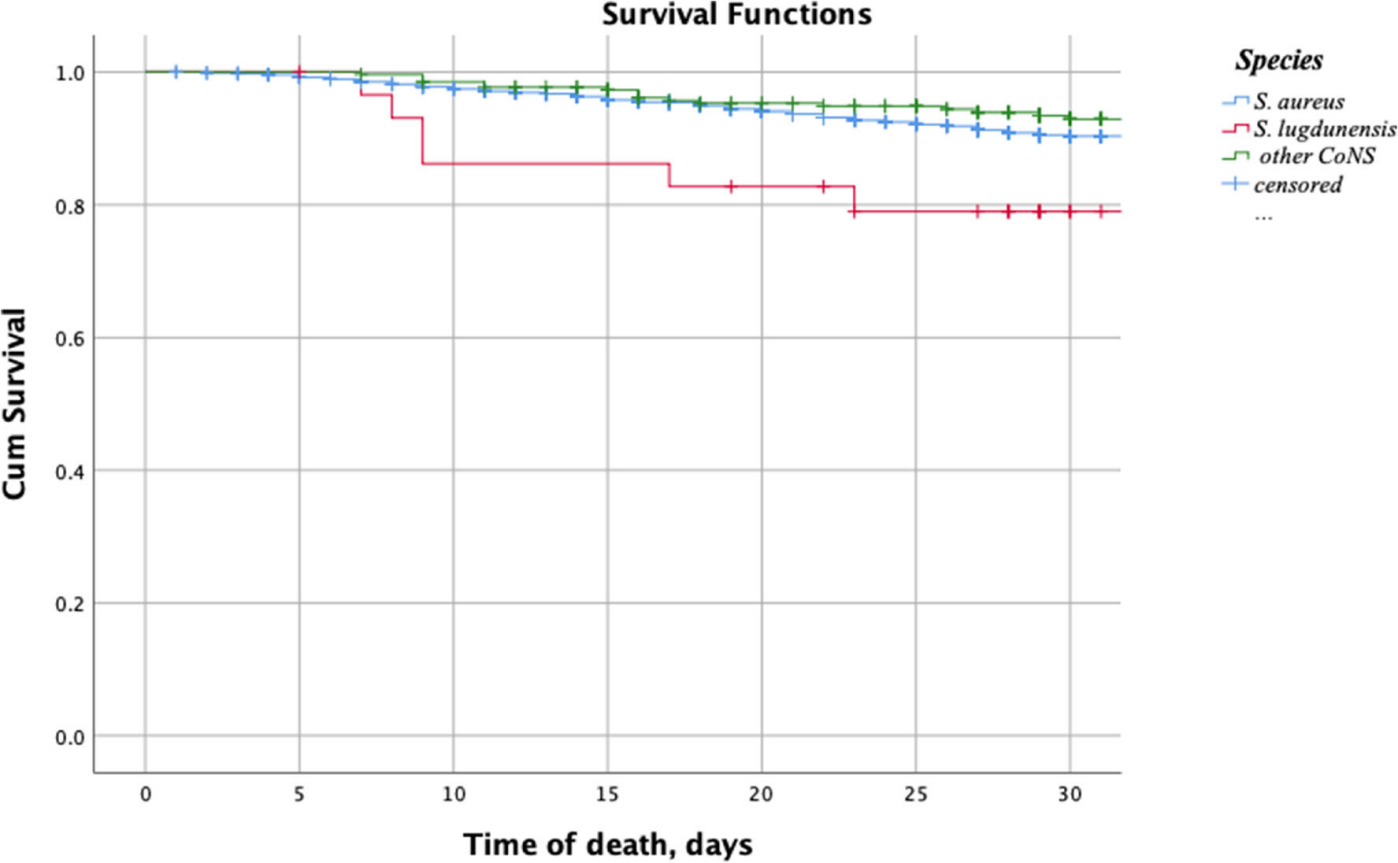
Kaplan-Meier plot survival after hospitalisation with IE caused by different staphylococci

In-hospital mortality was comparable between the groups. However, death occurred after a median time of 9 days in the *S. lugdunensis* group, which was significantly earlier than both the groups of *S. aureus* (median 25 days, *p* = 0.016) and other CoNS (median 36 days, *p* = 0.007) as visualised in Fig. 1 and Table 1. This makes all-cause mortality at 30 days significantly higher in the *S. lugdunensis* group (20%, *n* = 6) compared with other CoNS (7%, *n* = 17) and *S. aureus* (9%, *n* = 166), *p* = 0.016. This indicates, as previously reported, that *S. lugdunensis* on some occasions can cause an aggressive form of IE, which supports the importance of early identification and early surgical intervention for this pathogen [7, 8, 10].

The most common antibiotic treatment of *S. lugdunensis* IE, given to 18 cases (60%), was an isoxazolyl penicillin. All isolates were reported susceptible to isoxazolyl penicillin, except for two isolates where data were missing. Despite this, 7 patients (23%) received treatment with different antibiotic combinations, and only one of these patients had a prosthetic valve IE. Only one isolate was tested for penicillin G even though *S. lugdunensis* is known to have a conserved susceptibility to most antibiotics [5, 12, 20]. Recent research has suggested penicillin to be the preferred treatment of *S. lugdunensis* infections [12], and reliable methods for susceptibility testing are available [21].

The most important weakness of this study is the small number of *S. lugdunensis* IE cases. Given the few cases of *S. lugdunensis* IE, this study was underpowered to detect any small differences between the groups, and statistically significant differences have to be interpreted with caution. Even so, this is to our knowledge the largest *S. lugdunensis* IE cohort described, and the registry-based design of the study adds important knowledge about the clinical presentation of *S. lugdunensis* IE.

## References

[1] Freney J, Brun Y, Bes M, Meugnier H, Grimont F, Grimont PAD, Nervi C, Fleurette J (1988). *Staphylococcus lugdunensis sp*. nov. and *Staphylococcus schleiferi sp*. nov., two species from human clinical specimens. Int J Syst Evol Microbiol.

[2] Etienne J, Pangon B, Leport C, Wolff M, Clair B, Perronne C, Brun Y, Bure A (1989). *Staphylococcus lugdunensis* endocarditis. Lancet.

[3] Fleurette J, Bes M, Brun Y, Freney J, Forey F, Coulet M, Reverdy ME, Etienne J (1989). Clinical isolates of *Staphylococcus lugdunensis* and *S. schleiferi*: bacteriological characteristics and susceptibility to antimicrobial agents. Res Microbiol.

[4] Frank KL, Del Pozo JL, Patel R (2008). From clinical microbiology to infection pathogenesis: how daring to be different works for *Staphylococcus lugdunensis*. Clin Microbiol Rev.

[5] Anguera I, Del Rio A, Miro JM, Matinez-Lacasa X, Marco F, Guma JR, Quaglio G, Claramonte X, Moreno A, Mestres CA, Mauri E, Azqueta M, Benito N, Garcia-de la Maria C, Almela M, Jimenez-Exposito MJ, Sued O, De Lazzari E, Gatell JM, Hospital Clinic Endocarditis Study G (2005). *Staphylococcus lugdunensis* infective endocarditis: description of 10 cases and analysis of native valve, prosthetic valve, and pacemaker lead endocarditis clinical profiles. Heart.

[6] Patel R, Piper KE, Rouse MS, Uhl JR, Cockerill FR, Steckelberg JM (2000). Frequency of isolation of *Staphylococcus lugdunensis* among staphylococcal isolates causing endocarditis: a 20-year experience. J Clin Microbio.

[7] Sabe MA, Shrestha NK, Gordon S, Menon V (2014). *Staphylococcus lugdunensis*: a rare but destructive cause of coagulase-negative staphylococcus infective endocarditis. Eur Heart J Acute Cardiovasc Care.

[8] Kyaw H, Raju F, Shaikh AZ, Lin AN, Lin AT, Abboud J, Reddy S (2018). *Staphylococcus lugdunensis* endocarditis and cerebrovascular accident: a systemic review of risk factors and clinical outcome. Cureus.

[9] Koh TW, Brecker SJ, Layton CA (1996). Successful treatment of *Staphylococcus lugdunensis* endocarditis complicated by multiple emboli: a case report and review of the literature. Int J Cardiol.

[10] Farrag N, Lee P, Gunney R, Viagappan GM (2001). *Staphylococcus lugdunensis* endocarditis. Postgrad Med J.

[11] Frank KL, Reichert EJ, Piper KE, Patel R (2007). *In vitro* effects of antimicrobial agents on planktonic and biofilm forms of *Staphylococcus lugdunensis* clinical isolates. Antimicrob Agents Chemother.

[12] Taha L, Stegger M, Söderquist B (2019). *Staphylococcus lugdunensis*: antimicrobial susceptibility and optimal treatment options. Eur J Clin Microbiol Infect Dis: Off Publ Eur Soc Clin Microbiol.

[13] Kragsbjerg P, Bomfim-Loogna J, Törnqvist E, Söderquist B (2000). Development of antimicrobial resistance in *Staphylococcus lugdunensis* during treatment-report of a case of bacterial arthritis, vertebral osteomyelitis and infective endocarditis. Clin Microbiol Infect.

[14] Durack DT, Lukes AS, Bright DK (1994). New criteria for diagnosis of infective endocarditis: utilization of specific echocardiographic findings. Duke Endocarditis Service. Am J Med.

[15] Li JS, Sexton DJ, Mick N, Nettles R, Fowler VG, Ryan T, Bashore T, Corey GR (2000). Proposed modifications to the Duke criteria for the diagnosis of infective endocarditis. Clin Infect Dis: Off Publ Infect Dis Soc Am.

[16] EUCAST (2019) EUCAST clinical breakpoint Table http://www.eucast.org/fileadmin/src/media/PDFs/EUCAST_files/Breakpoint_tables/v_9.0_Breakpoint_Tables.pdf. Accessed 201210

[17] Liu PY, Huang YF, Tang CW, Chen YY, Hsieh KS, Ger LP, Chen YS, Liu YC (2010). *Staphylococcus lugdunensis* infective endocarditis: a literature review and analysis of risk factors. J Microbiol Immunol Infect.

[18] Fernandez Guerrero ML, Gonzalez Lopez JJ, Goyenechea A, Fraile J, de Gorgolas M (2009). Endocarditis caused by *Staphylococcus aureus*: a reappraisal of the epidemiologic, clinical, and pathologic manifestations with analysis of factors determining outcome. Medicine (Baltimore).

[19] Zinkernagel AS, Zinkernagel MS, Elzi MV, Genoni M, Gubler J, Zbinden R, Mueller NJ (2008). Significance of *Staphylococcus lugdunensis* bacteremia: report of 28 cases and review of the literature. Infection.

[20] Hellbacher C, Törnqvist E, Söderquist B (2006). *Staphylococcus lugdunensis*: clinical spectrum, antibiotic susceptibility, and phenotypic and genotypic patterns of 39 isolates. Clin Microbiol Infect.

[21] Hagstrand Aldman M, Påhlman LI (2020). Evaluation of penicillin G susceptibility testing methods for *Staphylococcus lugdunensis*. J Antimicrob Chemother.

